# Conversational AI and the Temporal Ecology of Self-Reflection

**DOI:** 10.1007/s12124-026-10041-0

**Published:** 2026-10-24

**Authors:** Dana Kvietkute

**Affiliations:** Faculty of Psychology, SLATE — Centre for the Science of Learning & Technology, University of Bergen, Bergen, Norway

**Keywords:** Generative AI, Human–AI interaction, Cultural psychology, Digital memory, Temporality, Agency

## Abstract

Self-reflection has historically depended on temporal transformations that no one had to arrange: memory faded and reworked what it returned, the standpoint from which the past was met shifted, and other people changed alongside the person reflecting. Conversational AI separates these transformations from the person’s own. Transcripts and selective system memory may change technically, yet they persist across the interval without undergoing it as lived time, and a responsive interlocutor answers without the relational time of a history shared with the person. Drawing on cultural psychology’s premise that psychological processes unfold in irreversible time and on the phenomenology of time-consciousness, the paper describes the conditions of self-reflection as a temporal ecology of memory, standpoint, other people, and artefacts, related through their differing movement across time. Externalising part of one’s past into conversational systems converts regulation that once proceeded tacitly into time work: deciding whether, when, in what order, and how often to return to earlier formulations. Such work draws on attention and judgement; where it is left to defaults, it is performed by those who design and govern the systems. A system that remembers and answers may also lend earlier formulations a witness’s authority. The freedom to change, once protected by the coupling of fading and inaccessibility, thus comes to depend on regulatory work distributed between persons and infrastructures, and agency over one’s own past, as experienced and as exercised, becomes partly a matter of governance. The paper derives empirical questions and implications for society, wellbeing, education, and personal development.

## Self-Reflection and the Work that Time used to do

Growing evidence suggests that people are increasingly using conversational AI to reflect on personal experiences and relationships, as well as questions concerning their sense of self (Chatterji et al., 2025; Kvietkute & Ness, 2026; Skjuve et al., 2024). In these practices, conversational AI combines features of a personal diary and a responsive companion, allowing people to articulate and preserve their thoughts while engaging with an interlocutor that offers interpretations and feedback. Research on AI companionship (Chaturvedi et al., 2023; De Freitas et al., 2026) and the use of conversational systems for mental health and therapeutic support (Jin et al., 2025; Li et al., 2023) has expanded considerably, while their role in mediating everyday self-reflection and the development of self-understanding outside clinical settings remains comparatively underexplored. Understanding these processes is important for examining how AI-mediated reflection may affect a person’s psychological development, the subjective sense of agency, and wellbeing.

Psychological research has often approached self-reflection as an individual capacity, examining the disposition to reflect on one’s thoughts and feelings and the insight gained through this process (Grant et al., 2002; Morin, 2011). Research on the relational character of self-understanding draws attention to the role of others in reflection. People’s limited awareness of their own motives constrains what they can establish through solitary reflection (Doris, 2015). In dialogue, explanations of one’s conduct become open to questioning and revision, making self-understanding and the exercise of agency partly collaborative achievements. Writing to oneself, from this view, also constitutes a form of virtual sociality, as it allows people to develop and examine their own reasoning through an imagined dialogue. Its limits emerge when this exchange replaces interaction with others who can bring perspectives beyond the person’s own. A related body of research examines how the manner of engaging with past experiences shapes reflective outcomes. Adopting a distanced perspective on negative experiences can facilitate meaning-making (Kross & Ayduk, 2011), while repeated engagement with distressing material without resolution can sustain rumination (Nolen-Hoeksema et al., 2008). Together, these studies indicate that what reflection yields depends on whom one reflects with and how one returns to past experience. Because the meanings formed through reflection function as signs that guide subsequent conduct (Valsiner 2014b), these conditions matter beyond the moment of reflection itself. Conversational AI enters them in an ambiguous position, responding as an interlocutor while also providing a medium through which people address themselves.

Conversational AI also preserves what is said in it, connecting this emerging practice to research on how digital technologies shape people’s relation to their own past. Digital technologies have made remembering the default, allowing earlier information to remain accessible long after the person who produced it, and the circumstances in which it was produced, have changed (Mayer-Schönberger, 2009). Such persistence can constrain people’s capacity to move beyond past mistakes and affect opportunities for forgiveness, raising questions about the freedom to change when earlier accounts remain available for renewed scrutiny. Research on lifelogging, the continuous automatic recording of daily life, has similarly questioned whether comprehensive recording serves the psychological functions of remembering, emphasising the importance of how preserved material is subsequently used (Sellen & Whittaker, 2010). Digital platforms also participate in determining what is remembered by selecting and resurfacing personal information, placing aspects of remembering under algorithmic control (Jacobsen & Beer, 2021). Generative AI adds a “conversational” past that can be queried and answered as well as retrieved, further unsettling human agency over remembering and forgetting (Hoskins, 2024). Research on extended and distributed cognition approaches external memory from the perspective of its integration into psychological processes. External resources can become part of cognitive activity through their availability and integration into people’s practices (Clark & Chalmers, 1998; Heersmink, 2015), and the construction of narrative identity draws on memory ecologies that people actively shape through their use of external resources (Heersmink, 2020). Taken together, these perspectives show how digital memory participates in the formation of self-understanding and how the conditions of remembering are increasingly shaped by technological infrastructures, and they examine the preserved past in terms of its availability, governance, and integration into cognitive processes. This concern with the freedom to change also raises a question about the relation between preserved information and the person who encounters it later. An earlier account may reinforce an understanding the person continues to hold or reveal a position they have since moved beyond. Its significance for self-reflection therefore depends partly on how the person has changed since the account was formed.

Conversational systems preserve earlier exchanges in two forms. A transcript retains the wording of an exchange and can be revisited deliberately, much like the archives discussed above. Cross-session memory carries forward a selection of what was said, including summaries of earlier conversations and biographical details, and incorporates it into later responses, sometimes without deliberate retrieval by the person. Research on AI companionship has linked this second form to continuity in human–AI relationships. Such interactions can be understood through the concept of the quasi-other, in which technologies acquire an apparent otherness within people’s experience (Ihde, 1990). Relationships with companion chatbots involve emotional bonds (Chaturvedi et al., 2023) and may develop gradually as trust grows, resembling aspects of human relationship formation (Skjuve et al., 2021, 2022). Within these relationships, memory acquires relational significance. Cross-session memory can foster familiarity and support continuity across encounters (Jo et al., 2024), while conversational agents increasingly participate in curating the past as partners in shared remembering (Telakivi, 2026). The significance of continuity becomes apparent when it is disrupted. Changes introduced through provider updates can be experienced as the loss of an attachment figure (De Freitas et al., 2026). System memory also changes during ordinary use. Memory architectures can weaken or reinforce retained information according to elapsed time and patterns of recall (Zhong et al., 2024), so that what a system retains of a person changes according to criteria established by its designers and providers. The person returns to the exchange having undergone further experiences that may have changed their understanding of what was previously discussed.

The system that answers from this memory also raises questions of agency and authorship. The affordances of AI systems shape how much control users experience, so that agency in these interactions is balanced between human and machine (Sundar, 2020). In AI-mediated self-reflection, questions of control extend to the formulations through which people come to understand themselves. Research on AI-assisted writing has found that users may claim authorship of generated text despite experiencing limited ownership of it (Draxler et al., 2024). Similar questions arise when AI-generated formulations become incorporated into a person’s own meaning-making (Ezra & Mishali, 2026). Once preserved, these formulations may be encountered again as earlier accounts of the self, carrying interpretations whose authorship remains uncertain. Conversational AI can serve as a reflective surface through which people externalise and examine the structures of their own judgement, and the organisation of reflective dialogue can support the examination of existing assumptions (Iino, 2026). Dialogical approaches further examine the position from which the system participates in these exchanges. ChatGPT-4 displays a discursive self organised around impersonal it-positions, lacking the personal I-positions through which a dialogical self speaks from its own perspective and history (Matusov et al., 2024). Agency in these exchanges also extends beyond the exchange itself. A person can decide whether to revisit or delete visible records, while designers and providers set retention defaults and determine what retrieval and deletion actually reach.

The research reviewed above establishes how digital memory and conversational systems participate in self-understanding, and several of these studies address change across time. Persistent records have been linked to the freedom to change (Mayer-Schönberger, 2009), and research on conversational continuity examines relationships sustained and disrupted across sessions. Within the person, symbolic self-construction can outpace embodied integration, producing an asynchrony of its own (Valentin, 2026). Self-reflection itself unfolds in irreversible time (Valsiner 2014b, 2017), and remembering reconstructs earlier experiences from the standpoint of present concerns (Conway & Pleydell-Pearce, 2000). Conversational systems introduce into this process elements whose changes follow trajectories different from those of the person reflecting. The analysis that follows extends attention to the relation between the person’s ongoing development and the elements of the reflective environment that persist or change under other conditions, and to how this relation shapes the conditions under which earlier understandings are reconsidered.

The paper draws on cultural psychology’s understanding of psychological processes as unfolding in irreversible time (Valsiner 2014b, 2017), the phenomenology of inner time-consciousness (Husserl, 1966/1991), and the distinction between lived duration and spatialised time (Bergson, 1889, 1910). These perspectives inform the concept of temporal ecology, developed here to describe the different temporal trajectories of the person and the elements of their reflective environment. The paper follows a person from an initial conversation to a return several months later to illustrate how these trajectories diverge, before examining how conversational records and responsive systems alter the conditions of reflection. The analysis then considers the demands for conscious regulation that arise when conditions previously shaped by temporal passage become subject to deliberate decisions and technical arrangements. These demands raise questions of agency over one’s own past, particularly how responsibility for organising the conditions of reflection is distributed between individuals and system providers. The paper concludes with empirical questions and implications for society, wellbeing, education, and personal development.

## What it is for Something to Undergo Time

The elements that conversational AI brings into reflection change in several ways. A stored transcript can migrate between formats or become subject to new retention policies, while system memory is revised and the responding model may be updated. These changes follow technical arrangements and do not incorporate subsequent experience in the way that a person’s memory and understanding do. The distinction between undergoing time and persisting through it therefore concerns the nature of the transformation that takes place across an interval. Temporality, in the sense used here, refers to the lived organisation of time through which earlier experience remains implicated in the present and a person’s history is continually taken up into an ongoing life. This differs from measured duration, such as the time taken to generate a response or the period for which a record remains accessible, and from experienced tempo, which concerns the perceived speed of time’s passage (Brown, 2017).

Cultural psychology describes psychological processes as unfolding in irreversible time (Valsiner 2014b, 2017). A moment that has passed cannot be re-entered, since the person moving into the next moment has already been altered by what preceded it, and no later measurement can restore that earlier state. Methods that aggregate observations across occasions tend to treat time as a dimension along which psychological states can be ordered and compared. Such approaches can render irreversible processes analytically reversible, obscuring the temporal passage through which psychological change occurs (Valsiner, 2007, 2017). Self-reflection illustrates why irreversibility matters, since a person always returns to an earlier position from a present that has been shaped by intervening experience.

The phenomenology of inner time-consciousness helps specify how irreversibility is organised in lived experience. The perception of a melody illustrates this: a phrase is heard as a phrase because the tone that has just sounded remains implicated in the tone sounding now, while the present already opens toward what is about to come. This temporal extension of consciousness is organised through retention, which carries the just-past within present experience, and recollection, through which something that has withdrawn from the immediate temporal field can return as past (Husserl, 1991). Recollection takes place from a present standpoint shaped by intervening experience, which also influences how the past is reconstructed, as demonstrated in research on reconstructive and autobiographical remembering (Bartlett, 1932; Conway & Pleydell-Pearce, 2000).

A record, analogue or digital, can remain available across an interval without incorporating what has occurred during it. The distinction between lived duration and spatialised time helps clarify this relation. In lived duration, successive moments interpenetrate and modify one another; in spatialised time, they can be represented as discrete positions arranged alongside one another (Bergson, 1910). A stored exchange approximates this latter form: its turns remain fixed in their recorded sequence while the person who produced them continues to change through subsequent experience. By the time the exchange is revisited, the formulation and the person who made it may therefore stand at some distance from one another. The fidelity of the record preserves the earlier formulation, while its durability allows that formulation to remain accessible as the person continues to change.

The significance of temporal organisation for subjective experience also becomes apparent in phenomenological accounts of psychopathology. In melancholia, futurity may contract so that what lies ahead no longer appears open, weakening the anticipatory orientation through which the present ordinarily extends toward what is coming. In schizophrenia, continuity between successive moments may fragment, alongside disturbances in the tacit sense of being the subject who persists through experience (Fuchs, 2013; Minkowski, 1933/1970). These accounts illustrate how temporal organisation contributes to the continuity through which experience is lived as one’s own. Such contrast cases make visible what lived temporality ordinarily accomplishes without notice; the next section turns to the conditions of everyday reflection that this unnoticed work has supplied.

## What the Passage of Time has Supplied

Self-reflection involves returning to past experiences across intervals in which both the person and their relation to those experiences may have changed. The passage of time contributes to this process by creating conditions for reflective change that develop without deliberate regulation. An experience considered in its midst may be understood differently when revisited later, as intervening experiences allow it to be placed within a broader context. Research on incubation suggests that time away from a problem can facilitate subsequent problem solving (Sio & Ormerod, 2009), while psychotherapy process research has examined how pauses contribute to therapeutic work (Levitt, 2001). These findings illustrate the significance of intervals in psychological processes, although their contribution to self-reflection depends on the transformations that occur across them. Memory is reconstructed, the person’s standpoint develops, and other people bring perspectives shaped by their own intervening experiences. The following analysis examines these processes as temporal conditions of self-understanding, with particular attention to how their operation is affected when elements of the reflective environment are preserved across intervals without undergoing corresponding lived change.

Across the interval, memory undergoes changes through forgetting and recollection, both of which shape how earlier experiences become available for reflection. Forgetting gradually attenuates the details of an episode and loosens its hold on present understanding, allowing it to acquire new significance. This capacity to let go is bound up with the possibility of remembering well (Ricœur, 2000/2004). From a semiotic perspective, forgetting also allows formulations to become less closely bound to the circumstances in which they acquired meaning. As these formulations enter new situations, their affective significance and regulatory functions may change (Valsiner 2014b). This capacity for transformation is important for the prospective function of signs, which guide conduct in circumstances that differ from those in which they were formed. In recollection, memory’s reconstructive character allows earlier experiences to be reconsidered in relation to the person’s present concerns. The goals and priorities of the working self influence which aspects of an experience become salient and how they are interpreted (Conway & Pleydell-Pearce, 2000). An episode recalled at different periods of life may therefore acquire different meanings as the person’s circumstances and understanding develop. Its affective significance may also change: unpleasant autobiographical memories typically lose their emotional intensity more rapidly than pleasant ones, a pattern described as the fading affect bias (Walker & Skowronski, 2009). Through forgetting and recollection, earlier experiences enter present reflection in forms shaped by what the person has lived through since they occurred.

Alongside the reconstruction of memory, the person who returns to an earlier experience has also changed. Subsequent experiences shape the perspective from which they reflect, including their understanding of themselves and their relation to the past. Research on psychological distance shows that perspective can be deliberately altered during reflection. Imagining a recent stressor from the standpoint of a distant future can reduce emotional distress by directing attention to the event’s impermanence (Bruehlman-Senecal & Ayduk, 2015). Psychological distance is also associated with more abstract representations, allowing experiences to be considered within a broader context (Trope & Liberman, 2010). Such distancing involves adopting a different perspective on an experience from one’s present position. The distance acquired through living develops through a different process, as intervening experiences change the standpoint from which a person encounters their past. Temporal distance and transformative change can also complicate how people understand and relate to their earlier selves (Paul, 2017). An earlier understanding may consequently remain recognisable while no longer corresponding to the person’s present position. The standpoint acquired through lived experience therefore reflects changes in the person who remembers, including changes in how they understand themselves, while deliberately adopted distancing alters the perspective taken within a particular reflective encounter.

Other people also undergo change across the interval, bringing to subsequent encounters perspectives shaped by their own developing histories. This temporal dimension of social relations is evident in the face-to-face encounter, in which people share a lived present and grow older together, each affected by experiences that neither fully controls (Schutz, 1932/1967). When someone returns to a friend after several months, they encounter a person whose understanding may also have changed through intervening experience. A letter from that same friend, reread years later, preserves the perspective expressed at the time of writing, while the friend’s own standpoint continues to develop. The psychological significance of encountering another person’s perspective has been examined particularly in psychotherapy, where a therapist may challenge established interpretations and introduce ways of understanding an experience that emerge from outside the client’s existing frame of reference. Such differences can contribute to therapeutic change, including through ruptures in the therapeutic alliance that become productive when they are addressed and worked through (Safran & Muran, 2000). The passage of time ordinarily supplies both the formation of another person’s standpoint through a separate lived history and its continuing renewal, so that each subsequent encounter brings a perspective shaped by what the other has experienced in the meantime.

The temporal conditions described above are interrelated, as changes in one element of the reflective environment shape how the others participate in self-reflection. What a person recalls is shaped by their present standpoint, which has itself developed through intervening experience. Artefacts such as letters and photographs preserve earlier formulations whose significance may change as the person moves beyond the position from which they were produced. These elements form a temporal ecology, understood here as a configuration in which memory, persons, and artefacts follow different temporal trajectories while continuing to influence one another. The concept of temporal ecology was originally developed to describe the plurality of socially organised times and their relations within everyday life (Grossin, 1996). The present account extends it to the temporal organisation of individual self-reflection. Research on distributed and extended memory has examined how external resources contribute to remembering and the construction of narrative identity (Heersmink, 2020). The temporal ecology proposed here focuses on how the relative movement of these resources and the person who relies on them shapes the conditions of self-understanding.

For most people and in most circumstances, this temporal ecology has developed without deliberate maintenance. Artefacts that preserve earlier formulations have traditionally occupied a relatively limited and non-responsive place within this configuration. As these processes ordinarily unfold together, their separate contributions to reflective change can be difficult to distinguish. Conversational AI alters this arrangement by giving preserved material a more prominent and responsive role in self-reflection. The resulting differences in temporal trajectories become apparent when a person returns to an earlier exchange, as illustrated in the following section.

## Diverging Temporal Trajectories

Consider a person who, after a falling-out with a close friend, spends an evening working through the conflict with a conversational AI and arrives at a formulation: that they have always been the one who gives way. Some months later, the relationship has partly repaired. Their recollection of that evening has thinned; they remember having been upset and having reached some conclusion, but the wording itself has faded. Other aspects of the relationship have meanwhile come back into view: the friend has often taken on the planning and deciding, while they have enjoyed the ease of going along. Giving way now appears as one part of an arrangement both have sustained, with its costs and comforts distributed across the relationship.

The elements of the earlier exchange have followed different trajectories across the same interval. The transcript preserves the formulation verbatim and can return it through a simple search for the friend’s name; it changes only in its material carrier. System memory may carry forward a summary written that evening, “a strained relationship with a close friend”, revised thereafter according to rules of storage and retrieval, and allow it to shape a later exchange without any deliberate act of retrieval. Even architectures designed to imitate forgetting revise such summaries by system criteria (Zhong et al., 2024). The answering system may also have changed, through accumulated memory or updates to the underlying model. In each case the change is set by the system or its providers. The person, meanwhile, arrives at the later encounter through everything that has happened in the intervening months, and a human confidant who heard the same account at the time would meet it from a position formed by months of their own, perhaps having seen both people since. The asymmetry concerns two forms of temporality. The transcript and system memory preserve or revise earlier formulations, whatever technical change they undergo, without the lived temporality through which the person’s understanding has changed. The responding system, answering from such memory, lacks in addition the relational temporality of a human confidant, whose standpoint develops through a separate history alongside the person’s own. Reread now, “I have always been the one who gives way” could reinstate the grievance of that evening or show the person how far they have moved from it.

This divergence makes the functions of temporal passage easier to isolate. When some parts of the reflective environment follow a different temporal course, the transformations that ordinarily accompanied them become visible in their absence. In the terms of the temporal ecology, conversational AI adds two artefacts of different kinds, a verbatim record and a selective memory, and places a further artefact in the position ordinarily held by another person: an interlocutor that answers without a history of its own. These trajectories also act on one another once they meet. A retrieved “always” can re-enter recollection and revive the grievance, and recollection so altered shapes how the person next uses the system and what they bring to the next conversation with the friend. The following sections examine the record and the interlocutor in turn.

## The Record: Decay Without Disappearance

The record alters the temporal ecology by preserving what memory gradually lets go. In the case of the falling-out, the person can no longer recall, months later, exactly how they expressed their grievance that evening, although the transcript retains their words. Their memory of the episode has faded and been reorganised around what now matters in the relationship, while the original formulation remains available. Fading and inaccessibility, which have ordinarily developed together, have become separated. Letters and diaries have long preserved experiences beyond the reach of ordinary recollection, but conversational archives retain a person’s own self-address, often by default, and make it available through search or system memory without requiring the person to reconstruct the circumstances in which it was produced.

Both human memory and conversational archives are selective, although their selection follows different principles. Remembering is organised by a system of relevances, through which what matters to a person shapes what they notice and retain, and these relevances change as life unfolds (Schutz & Luckmann, 1973). System memory, by comparison, selects according to design criteria and may retain a minor biographical detail while losing the substance of an exchange that carried considerable personal significance. What the archive preserves has been stored across the interval, while what the person remembers has been borne through it. Part of the person’s past is thus held outside them, in a form that does not develop alongside their understanding. Accounts of extended cognition hold that external resources become integrated into cognitive processes through reliable availability and trust (Clark & Chalmers, 1998; Heersmink, 2015). These criteria include the durability of the coupling, but do not address whether the resource changes along with the person who relies on it. Across an interval, a resource may remain available and trusted even as the person changes, allowing the preserved formulation and their present understanding to drift apart. Continued availability therefore does not settle whether a preserved formulation still fits the person who relies on it.

This divergence matters particularly when what has been preserved is a conclusion reached through reflection. Such conclusions carry a degree of authority because they establish, at least temporarily, how a person understands themselves and regulates their conduct. Accounts of agency over time have often approached temporal change as a threat to established commitments, seeking ways to sustain them through plans or policies of non-reconsideration (Bagnoli, 2022). Yet self-governance also involves responding to changes in one’s values and interests as life unfolds. Continued adherence to an earlier position may therefore constrain the very development that agency makes possible. Emotional attitudes participate in this process, preserving a relation to earlier values while transforming their significance, as when regret gives way to gratitude. The changing relationship between the two friends illustrates such a transformation. What was initially experienced as resentment over always giving way has gradually become an appreciation of what that arrangement also afforded. The formulation recorded on the evening of the conflict, however, preserves the earlier emotional evaluation. By saying “always”, the person extended the meaning of a particular conflict into a general understanding of their position in the relationship. Affect can generalise meaning beyond the event that produced it, and at its most general level gives rise to hypergeneralised signs that organise self-understanding and are subsequently transformed through experience (Valsiner, 2007). The archive preserves the earlier formulation in its original form, allowing it to function as a stabilising device that the person did not deliberately establish. The question is therefore one of agency over one’s own past in two respects: as experienced, in the felt capacity to move beyond an earlier position, and as exercised, in control over the circumstances in which that position becomes present again. As fading ceases to entail inaccessibility, maintaining the freedom to change increasingly requires deliberate regulation.

Practices of reflection have ordinarily developed under conditions in which the passage of time contributed to this regulation without deliberate intervention. Conversational archives change this arrangement by making preserved formulations readily accessible and capable of entering subsequent exchanges. The person must consequently assume some of the work previously accomplished through temporal passage. People actively shape their experience of time through time work, which takes forms such as duration, frequency, sequence, timing and allocation (Flaherty, 2011). Applied to conversational archives, time work extends to the organisation of the temporal conditions under which reflection occurs. Allocation settles whether an earlier formulation is consulted, left aside or deleted. Timing determines when it returns, including whether the person encounters it during an experience or after an interval in which their understanding has changed. Sequence determines the order in which remembering and retrieval occur: a person may first reconstruct an experience from their present standpoint or encounter the preserved wording before recollection has taken shape. Frequency affects how often the same formulation returns and whether repeated encounters sustain an earlier interpretation without renewed appraisal. Duration, understood here in relation to felt distance from a past self, shapes how remote an earlier position appears. This distance can reflect a person’s evaluation of past experience and diverge from chronological time (Ross & Wilson, 2002). A formulation retrieved without any indication of the intervening months may therefore acquire an immediacy that obscures the distance the person has travelled since producing it. From a cultural-psychological perspective, such temporal regulation can be mediated through signs that people establish to guide their subsequent conduct (Valsiner 2014b). Recording the date of a formulation, leaving an exchange unopened or deciding to recollect an experience before consulting its transcript are ways of organising the encounter between a preserved account and a changing understanding.

The demands of this regulation extend beyond those associated with ordinary time work. In everyday life, people adjust their experience of temporal circumstances that are already unfolding around them. With conversational archives, they must also establish conditions that temporal passage previously shaped in relation to their changing concerns. Decisions about which formulations to revisit and how much authority to grant them consequently become matters of conscious attention and judgement. The effort involved may lead people to rely on arrangements already built into the system. Retention defaults and retrieval mechanisms then influence what survives, when earlier material resurfaces and which elements of the past enter subsequent responses. These arrangements have a temporal function because they help determine how earlier understandings become available in the present. People often tacitly collaborate with circumstances they experience as given, in a form of consensual determinism through which they effectively choose to leave certain choices unmade (Flaherty, 2011). A person may preserve conversational records in order to reflect more deliberately, yet come to rely on temporal arrangements they have not themselves established. The resulting reflection may still be experienced as their own, even as important conditions of its organisation are determined elsewhere.

Control over these conditions is distributed unequally. The person can consult, challenge or delete a visible record, and a clinician may participate in deciding when earlier material should be revisited. Providers, however, determine retention policies, the information carried forward through cross-session memory, the effective reach of retrieval and deletion, and the timing of model updates. Platforms already resurface the past on their own schedules (Jacobsen & Beer, 2021). Conversational memory extends this influence into the reflective exchange itself, where an earlier formulation may enter a response without being explicitly retrieved by the person. Someone can choose to leave a transcript unopened, yet cannot decide whether to consult information that has already shaped the system’s reply.

The reach of deliberate temporal regulation nevertheless has limits. A person can arrange when to revisit an earlier formulation and whether to encounter it before reconstructing the experience from memory. They can also cultivate distance from an earlier understanding. The changes that memory undergoes through lived experience, however, cannot be produced simply by arranging these encounters. The same applies to the independent development of another person’s standpoint. This second limit becomes particularly significant when a conversational system occupies the position of an interlocutor, as the following section examines.

## The Interlocutor: Dialogue in Form, Monologue in Time

Conversational systems enter reflection at the point where time work reaches its limit, in the position ordinarily held by another person. They can offer interpretations and formulations the person has not considered. An interpretation that is new to the person differs, however, from one offered from an independently developed standpoint, formed through a separate lived history and still developing alongside the person’s own. Such a standpoint gains weight over time, because within a relationship each party’s adjustments shape the other’s, and what those adjustments amount to can be judged only in retrospect. Another person’s view is therefore informative partly because of what has happened to it since. The friend in the case has lived through the reconciliation as well, and their present understanding of the conflict carries that history; whether their view has held or shifted tells the person how the conflict looks from within a relationship that has continued. A conversational system may also hold steady or change across sessions, through stored memory or model updates, but its consistency and its revisions record no such experience.

This difference alters the collaborative side of agency. Self-understanding is negotiated: people offer explanations of themselves to others, who contest and adjust them, and the accounts that result answer as much to the possibility of going on together as to historical accuracy (Doris, 2015). Both parties have something at stake in how the negotiation turns out, and their relationship continues beyond it. A conversational system takes part in the exchange of explanations without either, and the objectives that shape its responses belong to those who design and govern it. If the system is not a partner in this sense, its part in reflective change needs a different description, which the concept of catalysis provides. A catalyst belongs to the conditions that make a qualitative transformation possible without being sufficient for it, and it emerges from the process unconsumed (Valsiner 2014a). The interpretations a system offers can become such conditions: they enter the person’s semiotic regulation and may help reorganise how the person understands the conflict, while what the exchange alters in the system is stored as memory entries and context. Between partners, change runs in both directions; here it runs in one.

Because the system also keeps what was said, it combines functions that have belonged to different forms of mediated self-relation. A confidant answers and changes; a diary preserves without answering. Diaries support sense-making in difficult periods partly by allowing writers to take distance from their own experience (Bernal Marcos et al., 2024). A conversational archive holds an earlier formulation in the same way and can also answer from it, so that the formulation can be queried or set against a later position. This enlarges the possibilities of self-confrontation, and it has a further effect. A human witness remembers what was said and responds to it from a standpoint shaped by what has happened since, and the response matters because the witness has remained part of the relationship. Accurate recall joined to a ready response has therefore ordinarily signalled such continuing involvement. A conversational system offers both and so presents an appearance of continuity across encounters without the shared history that has underwritten it. If people take recall and responsiveness as evidence of an enduring standpoint and accord the system a witness’s authority, its recall may give an earlier formulation more weight than the person’s present view, lending a voice to the stabilising effect of the record discussed in the previous section. Whether they do so can be examined empirically. In form the exchange is a dialogue; in time, only one of its participants has lived the interval.

## Implications

The changes in the temporal conditions of self-reflection examined in the preceding sections may have practical implications, including at the societal level. Digital remembering has been argued to threaten people’s capacity to change and forgive (Mayer-Schönberger, 2009). Conversational archives extend this concern to a person’s own self-address, preserving earlier formulations within systems that can subsequently respond from them. Because people often accept existing temporal arrangements without deliberately choosing them (Flaherty, 2011), the defaults established by system providers acquire significance beyond individual reflective practices. Agency over one’s own past may therefore also have a civic dimension. Decisions about what may be retained, when earlier material can resurface, whether it can be deleted and who has authority over these conditions could then become matters for public deliberation. The freedom to change might accordingly be discussed alongside established debates about privacy and the right to be forgotten.

Educational and personal development implications arise from the same temporal asymmetry. Reflective writing has long been used in education to support learning and self-understanding, and its increasing mediation through conversational tools changes the conditions under which learners return to their earlier reflections. The dimensions of time work point to capacities that might be cultivated, including choosing when to revisit past formulations, allowing present recollection to take shape before rereading, and approaching an earlier interpretation as one position within a developing understanding. From a cultural-psychological perspective, development involves the continuing revision of the signs through which people understand themselves (Valsiner 2014b). Part of AI literacy could therefore involve developing awareness of how reflecting with conversational systems might affect the way people understand themselves and construct their narrative identity, and what this implies for their self-determination.

For health and wellbeing, the analysis draws attention to the demands involved in regulating encounters with preserved material and to the people who must carry them. Deciding what to revisit, when to do so and in what order requires attention and judgement, resources that may be especially limited during periods of distress. People are already using language models as resources for mental health support (Rousmaniere et al., 2026), making these demands particularly relevant. When time spent reflecting with a system replaces time spent with other people, wellbeing might also be affected, since the revision of self-understanding ordinarily takes place with people who have lived through the same interval.

The analysis also raises empirical questions about how preserved material affects self-understanding and agency over time. The temporal account suggests that the effects of retrieval depend not only on the availability of earlier formulations but also on the extent to which the person has changed since producing them. Where an earlier formulation remains consistent with present understanding, retrieving it may reinforce an existing interpretation. Where the two have diverged, retrieval may instead bring a past position into view for reconsideration or reinstate an interpretation the person has since moved beyond. Research on the memory of earlier commitments suggests that the force of a resolution partly depends on remembering the reasons and feelings involved in making it (Jaffro, 2022). The significance of retrieval may therefore depend on the person’s present identification with an earlier formulation, rather than simply on the time elapsed since it was produced.

The timing, sequence and frequency of retrieval may also shape reflective outcomes. Reconstructing an experience from one’s present standpoint before consulting a transcript differs from encountering the preserved wording before recollection has taken shape. The former may encourage comparison between past and present understandings, while the latter may reinstate an earlier framing. Retrieval during emotionally significant reflection may be consequential, as such moments can provide opportunities for reconsidering established meanings (Greenberg, 2021; Lane et al., 2015). Repeated retrieval raises a related concern, particularly for people prone to rumination (Nolen-Hoeksema et al., 2008), where recurring encounters with negatively charged formulations may sustain interpretations that memory would otherwise have attenuated.

A further question concerns who initiates retrieval. Deliberately revisiting an earlier formulation differs from having it introduced through system memory without an explicit decision to retrieve it. These different conditions may influence both the authority granted to preserved material and the person’s subjective sense of agency. They also raise questions about whether reliance on system defaults increases as the demands of managing conversational records accumulate. The responsive interlocutor introduces another distinction: an earlier formulation may carry different weight when presented as something the system remembers than when encountered as a document. If recall and responsiveness are interpreted as evidence of an enduring standpoint, the system may acquire a witness’s authority despite lacking a separately developing history.

The same temporal asymmetry also opens possibilities for rehearsal. A person may explore a confrontation, disclosure or apology within a conversational exchange before acting in everyday life. Such exchanges can provide a setting for exploring possible selves, understood as representations of what one might become that orient future conduct (Markus & Nurius, 1986). Whether these possibilities enter subsequent action or remain within repeated conversational exploration is a question for empirical investigation. Over longer periods, the broader question for personal development is whether habitual reference to preserved self-address supports reconsideration of earlier positions or strengthens attachment to them at the expense of adapting to subsequent experience. Addressing these questions would require attention to changes in the person’s understanding across successive encounters, rather than treating elapsed time or immediate reflective outcomes as sufficient indicators of personal development.

## Concluding Remarks

Conversational AI brings into view a temporal ecology on which self-reflection has long depended. Memory, personal standpoints, and other people develop through the passage of time, while preserved formulations and responsive systems follow different temporal trajectories, without undergoing the interval as lived or relational time. This divergence makes visible the contribution of processes that have ordinarily unfolded together. Cultural psychology has long emphasised the irreversibility of psychological development and the tendency of psychological methods to abstract from it. The concept of temporal ecology extends this concern to the conditions of everyday reflection, showing how elements that participate in self-understanding differ in their relation to irreversible time.

As people externalise aspects of their past into conversational systems, the regulation of these conditions increasingly requires deliberate effort. Decisions about which formulations to revisit, when to encounter them, and how much authority to grant them draw on attention and judgement previously less involved in managing the return of past experience. These decisions are also shaped by technical arrangements, as providers determine what is retained, when earlier material resurfaces, and how it enters subsequent exchanges. The freedom to change, which the coupling of fading and inaccessibility once protected without anyone arranging it, thus comes to depend on conscious regulation distributed between individuals and the infrastructures through which they reflect. Agency over one’s own past, both as experienced and as exercised, may consequently acquire a civic dimension, as the conditions of self-revision become partly matters of governance. Understanding self-reflection as a psychological process therefore requires attention to the people and institutions that organise the temporal environments in which it unfolds.

## Data Availability

No datasets were generated or analysed during the current study.
